# Do pediatricians manage influenza differently than internists?

**DOI:** 10.1186/1471-2431-8-15

**Published:** 2008-04-24

**Authors:** Michael B Rothberg, Aleta B Bonner, MH Rajab, Barbara W Stechenberg, David N Rose

**Affiliations:** 1 Department of Medicine, Baystate Medical Center, Springfield, MA, USA; 2 Department of Medicine, Tufts University School of Medicine, Boston, MA, USA; 3 Department of Emergency Medicine, Scott and White Hospital, Temple, TX, USA; 4 Texas A&M University College of Medicine, Temple, TX, USA; 5 Department of Psychiatry, Scott and White Hospital, Temple, TX, USA; 6 Department of Biostatistics, Scott and White Hospital, Temple, TX, USA; 7 Division of Pediatric Infectious Diseases, Department of Pediatrics, Baystate Medical Center, Springfield, MA, USA

## Abstract

**Background:**

Little is known about how pediatricians or internists manage influenza symptoms. Recent guidelines on antiviral prescribing by the Centers for Disease Control and Prevention (CDC) make almost no distinction between adults and children. Our objective was to describe how pediatricians in two large academic medical institutions manage influenza and compare them to internists.

**Methods:**

At the end of the 2003–4 influenza season, we conducted a cross sectional on-line survey of physician knowledge, attitudes and practices regarding rapid diagnostic testing and use of antiviral therapy for influenza at two large academic medical centers, one in Massachusetts and the other in Texas. We collected data on self-reported demographics, test use, prescribing practices, and beliefs about influenza and anti-influenza drugs.

**Results:**

A total of 107 pediatricians and 103 internists completed the survey (response rate of 53%). Compared to internists, pediatricians were more likely to perform rapid testing (74% vs. 47%, p < 0.0001), to use amantadine (88% vs. 48%, p < 0.0001), to restrict their prescribing to high-risk patients (86% vs. 53%, p < 0.0001), and to believe that antiviral therapy could decrease mortality (38% vs. 22%, p = 0.01). Other beliefs about antiviral therapy did not differ statistically between the specialties. Internists were more likely to be unfamiliar with rapid testing or not to have it available.

**Conclusion:**

Pediatricians and internists manage influenza differently. Evidence-based guidelines addressing the specific concerns of each group would be helpful.

## Background

Influenza occurs in winter epidemics, affecting 5–20% of the population each year [1]. Children account for the majority of cases, but most of the morbidity and mortality occurs in elderly and other high-risk adults. Diagnosis can be made on clinical grounds [2-4] or by the use of rapid tests, which are widely available [5]. If diagnosed within 48 hours, 4 antiviral medications can be used to shorten the course of illness and prevent secondary infections requiring antibiotics [6]. There are no comparative trials, and most of the studies include only healthy adults; there are few studies of children [7-12]. Even less is known about high-risk patients. Based on the available data, the efficacy in children and adults appears similar, though high-risk adults may benefit more than either group [13]. Guidelines issued by the Centers for Disease Control and Prevention (CDC) in 2006 [14] do not distinguish between adults and children in their recommendations, except to point out that oseltamivir is licensed only for children aged ≥ 1 year and zanamivir for children aged ≥ 7 years. There are also no specific recommendations on whom to test.

The influenza epidemic of 2003–2004 was marked by its severity and the large number of pediatric deaths, which garnered significant media attention [15]. In the absence of official treatment guidelines, we hypothesized that pediatricians and internists would manage influenza differently, because complication rates and medication side effects differ between pediatric and adult populations.

## Methods

### Setting

Baystate Health, comprised of a 650-bed tertiary care center, 3 affiliated neighborhood health centers and a 330-physician multispecialty practice, provides primary and tertiary care to residents of Western Massachusetts. Baystate also serves as the western campus of the Tufts University School of Medicine. Scott & White Hospital and Clinic is located in Temple, Texas. It is comprised of a 550-physician multi-specialty group practice, a 503-bed tertiary care hospital and a self-contained Health Plan. Scott and White serves as the primary teaching hospital for the Texas A&M University College of Medicine.

### Study Design

We conducted a cross-sectional study of physician knowledge, attitudes and practices surrounding rapid diagnosis and antiviral therapy for influenza. The methodology has already been described [16]; this report focuses on differences between pediatricians and internists. During March and April of 2004 we sent direct e-mail invitations to members of the departments of pediatrics (n = 101) and medicine (n = 148) at Baystate Medical Center and the departments of pediatrics (n = 49) and medicine (n = 97) at Scott and White Hospital, inviting them to participate in an on-line influenza survey. Subjects were excluded if they did not have a valid e-mail address or did not care for patients with influenza.

Data were collected using a self-administered 41-question web-based survey (see Additional file 1), devised by the investigators. The survey contained questions about demographics (specialty, degree, years-in-practice and practice size), antiviral prescribing (numbers of prescriptions of various drugs, target groups and reasons for not prescribing), rapid testing (use of the test, choice of test and reasons for not testing) and influenza beliefs. We sent an initial e-mail explaining that the purpose of the survey was to study their knowledge, attitudes and practices regarding influenza testing and treatment. Anonymity was assured and completion of the survey implied informed consent. The study was approved by the institutional review board at each hospital. The survey ended on June 15, 2004.

### Statistical Analysis

The data were entered automatically into an electronic database by the internet survey company (surveymonkey.com), then downloaded into SAS version 9.1.3 (SAS Institute, Inc., Cary, NC). Responses were compared using the Chi-square or Fisher's exact tests, as appropriate. We created logistic regression models of each of the main study outcomes (prescribing antiviral therapy, prescribing amantadine, performing rapid testing and only treating high risk patients) controlling for specialty, location, patient volume, physician beliefs, and performance of rapid testing (for treatment outcomes only) using backward variable selection technique. P-values of less than 0.05 were considered statistically significant.

## Results

A total of 210 physicians in the two surveyed institutions completed the survey; 107 pediatricians and 103 internists (response rate of 54% for pediatricians and 52% for internists). Pediatricians reported that they were more likely to perform rapid testing than internists (73% vs. 46%, p < 0.0001), and more likely to treat with antiviral drugs, though the association did not reach statistical significance (Table 1). Pediatricians reported that they were also more likely than internists to use amantadine (88% vs. 48%, p < 0.0001), but not oseltamivir, and to restrict their prescribing to high-risk patients (86% vs. 53%, p < 0.0001). Differences in beliefs about the ability of antiviral therapy to shorten the course of illness and to prevent complications requiring antibiotics were not statistically significant, but more pediatricians than internists reported that they believed antiviral therapy could decrease mortality (38% vs. 22%, p = 0.01).

**Table 1:** Differences in practices and beliefs by specialty

	Pediatricians	Internists	P-value
	N (%) 107	N (%) 103	
**Actions**			
Performs rapid testing	78 (73)	47 (46)	< 0.0001
Prescribes antiviral drugs	63 (59)	49 (48)	0.1007
Prescribes amantadine	56 (88)*	23 (48)*	< 0.0001
Prescribes oseltamivir	25 (41)*	25 (52)*	0.2282
Treats only high risk	54 (86)*	26 (53)*	0.0001
			
**Beliefs**			
Shortens illness by 1 day	96 (90)	86 (84)	0.1847
Prevents complications	29 (27)	30 (29)	0.7443
Prevents hospitalization	53 (50)	39 (38)	0.0884
Decrease mortality	41 (38)	23 (22)	0.0119
None of the above	3 (3)	6 (6)	0.2798

*Denominator is total number of physicians in that specialty who prescribe antivirals.

**Figure 1:**
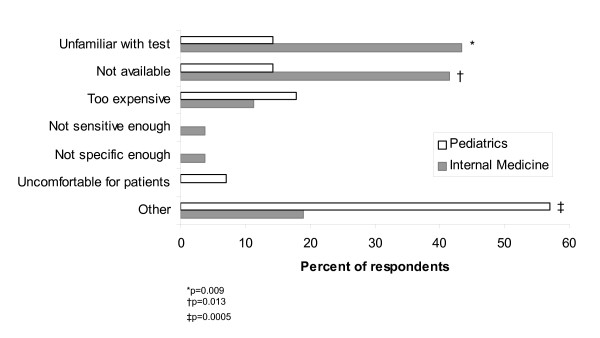
Reasons for not testing by specialty.

**Figure 2:**
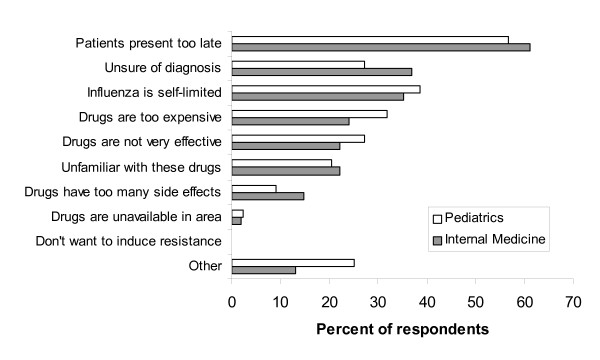
Reasons for not prescribing by specialty.

Reasons for not performing rapid testing and for not prescribing antiviral therapy are shown in Figures 1 and 2, respectively. Fewer pediatricians than internists reported that they were unfamiliar with rapid tests (14% vs. 43%, p = 0.009) or that the tests were not available (14% vs. 42%, p = 0.013). There was no statistical difference between specialties in their reasons for not prescribing antivirals. The most common reason reported was patients presented too late. Interestingly, no physician cited antiviral resistance as a reason for not prescribing.

In multivariate analysis, specialty remained significant for amantadine prescribing, rapid testing and restricting prescribing to high risk patients, but was not associated with antiviral prescribing overall (Table 2). Location, patient volume and the belief that antiviral therapy can decrease mortality were all associated with antiviral prescribing, but not with performance of rapid testing or restricting antiviral prescribing to high risk patients.

**Table 2:** Multivariate analysis for physician actions

Outcome	Variable*	Odds ratio	95% Confidence interval
Prescribes antiviral therapy			
	Location (MA vs. TX)	0.26	0.13 to 0.52
	Specialty (internists vs. pediatricians)	0.82	0.43 to 1.55
	Believes decreases mortality	1.6	1.4 to 5.7
	Low patient volume ( < 50 patients/week)	0.42	0.26 to 0.8
Prescribes amantadine			
	Specialty (internists vs. pediatricians)	0.18	0.06 to 0.49
	Believes decreases mortality	3.37	1.13 to 9.96
Performs rapid testing			
	Specialty (internists vs. pediatricians)	0.30	0.17 to 0.55
Treats only high risk patients			
	Specialty (internists vs. pediatricians)	0.19	0.07 to 0.54
	Believes decreases mortality	2.94	1.03 to 8.36

*All models include location, specialty, patient volume, physician beliefs, and performance of rapid testing. Only variables with significance of p < 0.05 are shown, except for specialty.

## Discussion

Our hypothesis that pediatricians and internists would differ in their management of influenza was borne out by the data. More pediatricians than internists were familiar with and performed rapid testing, perhaps because pediatricians are familiar with office-based rapid testing for respiratory syncytial virus and streptococcal pharyngitis. Indeed, internists' major reason for not performing testing was not being familiar with the test. Internists were significantly more likely to be unfamiliar with the test or to report that it was not available. Pediatricians may also perform rapid influenza testing in order to rule out sepsis in young children, based on the test's high specificity [17]. Although several decision analyses [18,19] have suggested that rapid testing is not cost-effective due to the low sensitivity of the test, particularly in adults [3], no pediatricians and fewer than 5% of internists cited low sensitivity as a reason for not testing.

Both specialties had similar beliefs about the efficacy of antiviral therapy and prescribed equally, but pediatricians preferred amantadine over oseltamivir. This preference may be influenced by the American Academy of Pediatrics Red Book, which states that the use of neuraminidase inhibitors "in the treatment of children requires further evaluation [20]," or it may reflect lower rates of amantadine side effects in children [21] or less detailing of oseltamivir to pediatricians. Because this study was conducted before the discovery of widespread amantadine resistance, it is unknown whether pediatricians have since decreased antiviral prescribing or simply converted to prescribing oseltamivir. Most pediatricians restricted their prescribing to high risk patients, while internists prescribed equally to high-risk and average-risk patients. This may have to do with pediatricians' belief that antiviral drugs could decrease mortality, or hesitancy on the part of pediatricians to trade off a reduction in symptoms versus potential side effects in low risk patients. In light of the recent reports of pediatric deaths in Japanese children treated with oseltamivir, such prudence may be warranted [22].

Our finding that 38% of pediatricians believe that antiviral drugs improve mortality is particularly interesting because there are no published antiviral studies that include high-risk children, and no antiviral study has ever been powered to detect a difference in mortality. On a similar note, more pediatricians than internists believed that antivirals could prevent hospitalization, despite the fact that this has only been demonstrated in adults [23].

Among those who did not prescribe antiviral therapy, both pediatricians and internists reported similar reasons for not prescribing, specifically that patients do not present soon enough. Although more than two-thirds of pediatricians and internists felt this to be true, a subsequent study of children with influenza-like illness found that 25% of outpatients and 42% of emergency department patients with true influenza present within 2 days, but that clinicians often miss the diagnosis [24]. Interestingly, no physician cited antiviral resistance as a reason for not prescribing antiviral therapy. At the time of the study, antiviral resistance was rare and the clinical significance unknown. Since then, however, amantadine resistance has become widespread [25] and resistance to oseltamivir has been growing [26].

Interim guidelines issued by the CDC for 2006 make almost no distinction between the treatment of children and adults. The guidelines stress treatment with oseltamivir due to high levels of amantadine resistance, with priority given to high-risk individuals where local supplies are limited. Presumably, where supplies are sufficient, treatment may extend to lower risk patients. However, no guidance is offered on how to determine who is infected, nor what factors to consider in deciding about treatment. Moreover, the guidelines offer no advice on overcoming the biggest obstacle to antiviral prescribing, namely, getting patients to the doctor within the 48-hour window during which antivirals are effective. Based on the results of our survey, adherence to the guidelines will require more of a change in practice by pediatricians, who generally prefer amantadine to oseltamivir. In light of the large, unrecognized burden of influenza in children [24], specific recommendations about rapid testing would also be welcome. Given the wide variation in practice demonstrated in this survey, more detailed, evidence-based guidelines, with attention to the differing concerns of pediatricians and internists, would be welcome.

Our study has several limitations. Respondents, limited to two academic medical centers, may not be representative of the nation as a whole. Moreover, the anonymous nature of the survey did not allow us to compare the demographics of responders and non-responders. Nevertheless, the observed differences were quite striking and not likely to have occurred by chance. Also, our survey was conducted before the emergence of widespread amantadine resistance. Consequently, prescribing practices may have changed, though beliefs and testing practices probably have not. Given that adults and children seem to benefit equally from influenza treatment [6], more detailed guidelines are needed to ensure a standardized approach to all patients.

## Conclusion

Despite that fact that adults and children appear to benefit equally from influenza diagnosis and treatment, pediatricians appear to differ from internists in their approach to influenza. Specifically, pediatricians are more likely to employ rapid testing, to limit treatment to high risk patients, to treat with amantadine and to believe that antiviral therapy can reduce mortality. In light of these practice differences, age-specific evidence-based recommendations regarding rapid diagnostic testing and antiviral therapy for influenza are warranted.

## Competing interests

MR has served as a paid consultant for Quidel Corporation and has received an honorarium from Roche. Dr. Bonner has served as a consultant for Quidel and Biostar, and has received grant funding from Quidel and Binax. No outside funding was accepted in the conduct of this research.

## Authors' contributions

MR conceived of the study, participated in its design, collected the data, and drafted the manuscript. AB participated in the study design, collected and interpreted the data. MHJ performed the statistical analysis. BS participated in the design of the study and interpretation of the data. DR participated in the design of the study and interpretation of the data. All authors read and approved the final manuscript.

## Pre-publication history

The pre-publication history for this paper can be accessed here:



## Supplementary Material

Additional file 1 Survey instrument Click here for file
