# Supplementary material for: Do pediatricians manage influenza differently than internists?
Source: BMC Pediatr. 2008 Apr 24;8:15. doi: 10.1186/1471-2431-8-15 (PMC2375866; doi:10.1186/1471-2431-8-15)
Supplement: Additional file 1 — Survey instrument [file 1471-2431-8-15-S1.doc]

This survey will assess your knowledge, attitudes and practices regarding testing and treatment of influenza. At the end, you'll see how your colleagues responded.

Please provide us with some background information on yourself and your practice.

| **1. What is your specialty?** |
| --- |
|  |

| 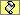[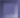](http://www.surveymonkey.com/SurveySummary.asp?SID=411967&Rnd=0.3581912##) |  | Internal Medicine |
| --- | --- | --- |
|  | |  |
| [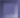](http://www.surveymonkey.com/SurveySummary.asp?SID=411967&Rnd=0.3581912##) |  | Family Practice |
|  | |  |
| [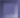](http://www.surveymonkey.com/SurveySummary.asp?SID=411967&Rnd=0.3581912##) |  | Pediatrics |
|  | |  |
| [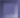](http://www.surveymonkey.com/SurveySummary.asp?SID=411967&Rnd=0.3581912##) |  | Infectious Disease |
|  | |  |
| [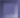](http://www.surveymonkey.com/SurveySummary.asp?SID=411967&Rnd=0.3581912##) |  | Emergency Medicine |
|  | |  |
| [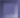](http://www.surveymonkey.com/SurveySummary.asp?SID=411967&Rnd=0.3581912##) |  | Other (please specify) |

| **2. What is your degree?** |
| --- |
|  |

| 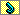[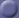](http://www.surveymonkey.com/SurveySummary.asp?SID=411967&Rnd=0.3581912##) |  | P.A. |
| --- | --- | --- |
|  | | |
| [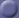](http://www.surveymonkey.com/SurveySummary.asp?SID=411967&Rnd=0.3581912##) |  | D.O. |
|  | | |
| [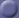](http://www.surveymonkey.com/SurveySummary.asp?SID=411967&Rnd=0.3581912##) |  | M.D. |
|  | | |
| [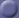](http://www.surveymonkey.com/SurveySummary.asp?SID=411967&Rnd=0.3581912##) |  | N.P. |

| **3. How long have you been in practice?** |
| --- |
|  |

| 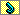[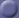](http://www.surveymonkey.com/SurveySummary.asp?SID=411967&Rnd=0.3581912##) |  | Resident |
| --- | --- | --- |
|  | | |
| [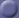](http://www.surveymonkey.com/SurveySummary.asp?SID=411967&Rnd=0.3581912##) |  | <5 years |
|  | | |
| [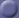](http://www.surveymonkey.com/SurveySummary.asp?SID=411967&Rnd=0.3581912##) |  | >=5 years |

| **4. Approximately how many patients do you see per week?** |
| --- |
|  |

| 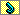[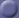](http://www.surveymonkey.com/SurveySummary.asp?SID=411967&Rnd=0.3581912##) |  | <50 |
| --- | --- | --- |
|  | | |
| [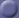](http://www.surveymonkey.com/SurveySummary.asp?SID=411967&Rnd=0.3581912##) |  | 50-100 |
|  | | |
| [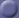](http://www.surveymonkey.com/SurveySummary.asp?SID=411967&Rnd=0.3581912##) |  | >100 |

| *** 5. How many times have you prescribed antiviral drugs for influenza this season?** |
| --- |
|  |

| 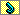[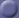](http://www.surveymonkey.com/SurveySummary.asp?SID=411967&Rnd=0.3581912##) |  | 0 |
| --- | --- | --- |
|  | | |
| [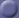](http://www.surveymonkey.com/SurveySummary.asp?SID=411967&Rnd=0.3581912##) |  | 1-5 |
|  | | |
| [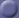](http://www.surveymonkey.com/SurveySummary.asp?SID=411967&Rnd=0.3581912##) |  | >5 |

| **6. How many prescriptions have you written for each of the following drugs this season?** |
| --- |
|  |

|  |  |  | 0 |  |  | 1-5 |  |  | >5 |  |
| --- | --- | --- | --- | --- | --- | --- | --- | --- | --- | --- |
| 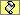 |  |  |  |  |  |  |  |  |  |  |
|  | | | | | | | | | | |
|  | Rimantidine (Flumadine) |  | [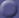](http://www.surveymonkey.com/SurveySummary.asp?SID=411967&Rnd=0.3581912##) |  |  | [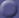](http://www.surveymonkey.com/SurveySummary.asp?SID=411967&Rnd=0.3581912##) |  |  | [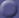](http://www.surveymonkey.com/SurveySummary.asp?SID=411967&Rnd=0.3581912##) |  |
|  | | | | | | | | | | |
|  | | | | | | | | | | |
|  | Zanamivir (Relenza) |  | [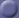](http://www.surveymonkey.com/SurveySummary.asp?SID=411967&Rnd=0.3581912##) |  |  | [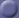](http://www.surveymonkey.com/SurveySummary.asp?SID=411967&Rnd=0.3581912##) |  |  | [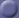](http://www.surveymonkey.com/SurveySummary.asp?SID=411967&Rnd=0.3581912##) |  |
|  | | | | | | | | | | |
|  | | | | | | | | | | |
|  | Oseltamivir (Tamiflu) |  |  |  |  |  |  |  |  |  |
|  | | | | | | | | | | |
|  | | | | | | | | | | |
|  | Amantadine (Symmetrel) |  |  |  |  |  |  |  |  |  |
|  | | | | | | | | | | |

| **7. For which groups of patients do you prescribe antiviral drugs for influenza (check all that apply)?** |
| --- |
|  |

|  |  | Children 1-5 years old |
| --- | --- | --- |
|  | |  |
|  |  | Children 6-18 years old |
|  | |  |
|  |  | Children with chronic medical conditions |
|  | |  |
|  |  | Elderly (>65 years) |
|  | |  |
|  |  | Heart disease |
|  | |  |
|  |  | Lung disease |
|  | |  |
|  |  | All Patients |
| **8. Why don't you prescribe antiviral drugs for influenza (Check all that apply)?** | | |
|  | | |

|  |  | Unsure of diagnosis |
| --- | --- | --- |
|  | |  |
|  |  | Don't want to induce resistance |
|  | |  |
|  |  | Influenza is self-limited |
|  | |  |
|  |  | Unfamiliar with these drugs |
|  | |  |
|  |  | Patients present too late for treatment |
|  | |  |
|  |  | Drugs are unavailable in my area |
|  | |  |
|  |  | Drugs are too expensive |
|  | |  |
|  |  | Drugs are not very effective |
|  | |  |
|  |  | Drugs have too many side effects |
|  | |  |
|  |  | Other (please specify) |

| **9. How many prescriptions have you written for an antibiotic for acute bronchitis this season?** |
| --- |
|  |

|  |  | 0 |
| --- | --- | --- |
|  | | |
|  |  | 1-5 |
|  | | |
|  |  | >5 |

| **10. Do you perform rapid diagnostic testing for influenza in your office?** |
| --- |
|  |

|  |  | Yes |
| --- | --- | --- |
|  | | |
|  |  | No |

| **11. Which rapid test do you use?** |
| --- |
|  |

|  |  | Quickvue |
| --- | --- | --- |
|  | |  |
|  |  | ZStatFlu |
|  | |  |
|  |  | Directogen A |
|  | |  |
|  |  | Directogen A+B |
|  | |  |
|  |  | FluOIA |
|  | |  |
|  |  | Now Flu A |
|  | |  |
|  |  | Now Flu B |
|  | |  |
|  |  | Not sure/ Don't know |

| **12. Do you send out samples for rapid diagnostic testing for influenza?** |
| --- |
|  |

|  |  | Yes |
| --- | --- | --- |
|  | | |
|  |  | No |
| **13. Which of the following keep you from using rapid tests for influenza (check all that apply)?** | | |
|  | | |

|  |  | Uncomfortable for patients |
| --- | --- | --- |
|  | |  |
|  |  | Not specific enough |
|  | |  |
|  |  | Not available |
|  | |  |
|  |  | Not sensitive enough |
|  | |  |
|  |  | Unfamiliar with test |
|  | |  |
|  |  | Too expensive |
|  | |  |
|  |  | Other (please specify) |

| *** 14. Which of the following do you believe to be true of at least one antiviral drug for influenza? (May check more than one)** |
| --- |
|  |

|  |  | Shortens the course of illness by one day |
| --- | --- | --- |
|  | |  |
|  |  | Prevents complications (e.g. sinusitis, otitis media, pneumonia) |
|  | |  |
|  |  | Prevents hospitalizations in high risk patients |
|  | |  |
|  |  | Decreases mortality |
|  | |  |
|  |  | None of the above |

Please feel free to comment on our survey!

Thanks for advancing medical knowledge! Click "Done" to see how others responded.
